# Misperceptions of "light" cigarettes abound: National survey data

**DOI:** 10.1186/1471-2458-9-126

**Published:** 2009-05-08

**Authors:** Nick Wilson, Deepa Weerasekera, Jo Peace, Richard Edwards, George Thomson, Miranda Devlin

**Affiliations:** 1 Department of Public Health, University of Otago, Wellington, Box 7343, Wellington South, New Zealand; 2 Health & Disability Intelligence, Ministry of Health, PO Box 5013, Wellington, New Zealand

## Abstract

**Background:**

Many smokers believe that "light" cigarettes are less harmful than regular cigarettes, which is at variance with the scientific evidence. The Framework Convention on Tobacco Control (FCTC) aims to address this problem in Article 11 which deals with misleading labelling of tobacco products. In this study we aimed to determine smokers' use and beliefs concerning "light" and "mild" cigarettes ("lights"), including in relation to ethnicity, deprivation and other socio-demographic characteristics.

**Methods:**

The New Zealand (NZ) arm of the International Tobacco Control Policy Evaluation Survey (ITC Project) uses as its sampling frame the NZ Health Survey. This is a national sample with boosted sampling of Maori, Pacific peoples and Asians. From this sample we surveyed adult smokers (n = 1376) about use and beliefs relating to "light" cigarettes. We assessed the associations with smoking "lights" after adjusting for socio-demographic variables, and smoking-related behaviours and beliefs.

**Results:**

Many smokers of "lights" believed that smoking "lights" made it easier to quit smoking (25%), that "lights" are less harmful (42%), and that smokers of "lights" take in less tar (43%). Overall most "lights" smokers (60%) had at least one of these three beliefs, a proportion significantly higher than for smokers of "regular" cigarettes at 45% (adjusted odds ratio (aOR) = 1.96, 95% CI = 1.29 – 2.96). While "lights" smokers had significantly lower tobacco consumption and were more aware of smoking harms, they were no more likely to be intending to quit or have made a previous quit attempt.

By ethnicity, both Maori and Pacific people were less likely to smoke "lights" than Europeans (aOR = 0.53, 95% CI = 0.35 – 0.80 and aOR = 0.14, 95% CI = 0.05 – 0.40 respectively). In contrast there was no significant difference by level of deprivation. Roll-your-own (RYO) tobacco smokers were less likely to smoke "light" forms of RYO tobacco while both older and women smokers were more likely to smoke "lights".

**Conclusion:**

Most "lights" smokers have one or more misperceptions about the product they use, and were no more likely to intend to quit or to have made a quit attempt. In response to such misperceptions, governments could act further to eliminate all misleading tobacco marketing. Ideally, they could not only adopt FCTC requirements, but go further by requiring plain packaging for all tobacco products.

## Background

"Light" or "mild" cigarettes (as defined by smoke machine testing) have been engineered to give low tar readings. For example, the filters in "light" cigarettes often include ventilation holes which allow air to enter and dilute the smoke. These result in machine-tested "light" cigarettes appearing to deliver less toxic constituents in the inhaled smoke than regular cigarettes. Furthermore, "lights" have been marketed to appeal to health concerned smokers, and positioned as an alternative to quitting [1].

However, there is evidence that "light" cigarettes often deliver as much tar as regular cigarettes in real smokers [2]. There are several reasons for this including that many smokers will block the ventilation holes with their fingers, preventing the dilution effect. Also many smokers who switch to "lights" from regular cigarettes appear to "compensate" for the lower nicotine levels by inhaling more deeply, taking nicotine-driven, larger, more rapid, or more frequent puffs; and by increasing the number of cigarettes smoked per day. As a result of these actions, smokers appear to cancel out any potential benefit of smoking a "light" cigarette over a regular one [2]. This is supported by toxicological and epidemiological evidence that suggests no significant health benefit in terms of lung cancer, heart disease or chronic lung disease for smoking "light" versus other cigarettes [2-5]. Even more concerning is that the marketing appears to work, in that there is evidence that smokers use "lights" as an alternative to quitting [6].

Despite this evidence, a number of international studies have found that many smokers believe that "lights" are less harmful than regular cigarettes [1,7-12]. These studies have been conducted in the US [1,7,8], Canada [8,11], Switzerland [9], the UK [8], and Australia [8]. These misperceptions appear to be deeply imbedded, and in the UK were only partially corrected by an information campaign and the removal of such descriptors from packets [13]. The Framework Convention on Tobacco Control (FCTC) aims to address this problem in Article 11 [14]. It requires ratifying countries to enact laws to ensure that:

"tobacco product packaging and labeling do not promote a tobacco product by any means that are false, misleading, deceptive or likely to create an erroneous impression about its characteristics, health effects, hazards or emissions, including any term, descriptor, trademark, figurative or any other sign that directly or indirectly creates the false impression that a particular tobacco product is less harmful. These may include terms such as 'low tar', 'light', 'ultra-light', or 'mild'."

At least 33 countries have responded to these issues and the FCTC provisions by prohibiting such descriptors on cigarette packages and in advertising eg, actions by the European Union, Australia, Brazil, Peru and Venezuela [15]. In some others (eg, Israel [16]), criminal investigations into the tobacco industry are reported to be underway or have found that the law has been violated, as in the case of the USA [17].

In New Zealand, there is limited local survey evidence that smokers believe that "light" cigarettes do less harm than regular cigarettes [18]. A study of popular brand variants of New Zealand cigarettes even found a significantly *higher *ratio of carbon monoxide per mg of nicotine in "light" versus regular cigarettes [19]. There also have been calls for action on misleading descriptors in New Zealand [20-22], and in late 2008 the Commerce Commission warned the tobacco industry over the use of misleading descriptors [23].

In this study we explored the hypothesis that misperceptions of "lights" existed in New Zealand and that these would vary by ethnicity, deprivation levels and roll-your-own (RYO) usage. The New Zealand population is well suited for such an investigation as it has marked variation in smoking by ethnicity and deprivation [24], and has relatively high levels of RYO usage (49% among Europeans and 60% among Māori [25]). Furthermore, the topic remains of continued relevance in that tobacco manufacturers (in New Zealand [22] and elsewhere) appear to be using colour-coding of cigarette packaging to perpetuate the "light" versus "regular" brand distinctions where the descriptor words such as "light" are restricted. Such considerations are therefore important in informing possible policy responses (such as plain packaging) and future revisions of FCTC provisions around packaging.

## Methods

The International Tobacco Control Policy Evaluation Survey (the ITC Project) is a multi-country study on tobacco use epidemiology and tobacco control policy evaluation. A full description of the ITC Project conceptual framework and methods have been published elsewhere [26,27] but to summarise, it uses a prospective multi-country cohort design and theory-driven mediational models that allow tests of hypotheses about the anticipated effects of given policies.

The New Zealand arm of the ITC Project survey differs somewhat from the other ITC Project countries in that the smokers involved are from the sample frame of New Zealand Health Survey (NZHS) participants (with this survey being conducted in 2006/2007). Methods of the NZHS are detailed more fully in the report on the key results [28] and a detailed methods report [29]. But to summarise, the NZHS is a general health survey of the New Zealand population of all ages. Respondents were selected by a complex sample design, which included systematic boosted-sampling of the Māori, Pacific and Asian populations. Interviews were conducted face-to-face in respondents' homes by trained interviewers (on contract to the Ministry of Health) and resulted in a total of 11,924 interviews with respondents aged 18 and over. The overall response rate was 67.9%. Other issues around the NZHS response rate as it relates to the ITC project are detailed in an online *Methods Report *[30].

### Participants

From the NZHS sample we had an additional sampling frame of adult smokers who had all of the following characteristics: (i) were 18 years or older; (ii) who had smoked more than 100 cigarettes in their lifetimes; (iii) who had smoked at least once in the past 30 days; and (iv) who had agreed to be willing to participate in further research when asked this at the end of the NZHS interview (this was 85.2% of the adult smokers in the NZHS). Out of 2438 potential respondents who met these criteria, a total of 1376 completed the NZ ITC Project Wave 1 questionnaire giving a response rate of 56.4%. If however, the smokers who were unwilling to participate in further research (when asked as part of the NZHS) are considered in the denominator, then this response rate is 48.0% (1376/2866). Other issues around the response rate are detailed in an online *Methods Report *[30].

Although participants were all smokers at the time of the NZHS, a small proportion had quit (Table 1), but this group were retained in the cohort (as per the general ITC Project approach and because smokers who have recently quit have high relapse rates).

**Table 1:** Characteristics of respondents in this sample (weighted sample with adjustment for complex sample design, n = 1376)

**Characteristic**	**(%)**
***Smokers not considered in the subsequent analyses***	
Smokers at the time of the NZHS who have since quit	12.0
Current smokers who did not describe a specified brand they usually smoked	6.0
	
***"Lights" smokers – considered in the subsequent analyses***	
Current smokers who usually smoke a brand of "light" cigarettes	13.0
Current smokers who usually smoke a brand of "light" tobacco (for roll-your-own (RYO) cigarettes, of which there are a number of brands on the NZ market)	5.7
	
***Regular smokers – considered in the subsequent analyses***	
Current smokers who usually smoke a regular brand of cigarettes	32.4
Current smokers who usually smoke a regular brand of tobacco (for RYO cigarettes)	30.5
	**100%**

### Procedures

Surveying of these participants was carried out using a computer-assisted telephone survey (sub-contracted to Roy Morgan Research). The first wave of participants were all interviewed between March 2007 and February 2008, usually 3–4 months after their NZHS interview. The study protocol was cleared by the Multi-Region Ethics Committee in New Zealand (MEC/06/07/071) and by the Office of Research Ethics, University of Waterloo, Waterloo, Canada (ORE #13547).

### Measures

The particular questions relating to the use and beliefs of "lights" were generally identical to those from the standard survey questionnaire for the ITC survey used in other countries (and as described previously by others [8]). Where differences occurred (due to these questions being included in the NZHS) these are reported in footnotes below the tables. For indices used we calculated scores for assessing internal consistency (Cronbach's alpha) and these indices were only used if the scores were at least 0.5.

For the tobacco products available in the New Zealand setting we defined "lights" as cigarettes or loose tobacco that had a brand name with any mention of one or more of the following words: "light", "mild", "blue", "silver", "gold", and "low-tar". We included these words for colours based on our background work that indicated that tobacco manufacturers for the New Zealand market had been recently changing brands with the descriptors "light", "mild" and "low-tar" to ones with the words for the colours of "blue", "silver" and "gold" [22]. Overall however, the classification we used was somewhat conservative (ie, favouring a "regular" classification over a "lights" classification) in that we did not include "lower" (ie, <16 mg) tar figures that were associated with particular brands when none of the words detailed in the list above were mentioned. This was because we considered that descriptor words on the label would have more psychological impact on smokers than tar levels in small print on the side of the pack. Also, only a minority of smokers appear to be aware of the reported tar levels for their cigarettes [31].

### Weighting and statistical analyses

Weighting of all the results to reflect the national population of smokers was necessary given the sampling design (eg, boosted sampling of three ethnic groups in the NZHS) and non-response for the NZHS and ITC Project survey. A full description of the weighting process is detailed in an online report [32].

Univariate analysis of all socioeconomic and smoking variables was initially conducted (Tables 2, 3, 4 and 5). We also carried out a multivariate logistic regression analysis (Table 6). This analysis used a conceptual framework which assumed that there would be hierarchical relationships between demographic and socio-demographic factors [33], that would dominate over smoking-related behaviours and beliefs. All models included age, gender and ethnicity and models 2–4 included key socio-demographic variables (ie, deprivation). For the other models we entered variables relating to smoking behaviour (model 3) and smoking-related beliefs (model 4). For models 3 and 4, variables were selected with a p-value of < 0.05 in the univariate analyses and a forward selection procedure was used to select the final model with only statistically significant smoking-related behaviour and belief variables retained in the final models (as detailed in Table 6). All analyses were conducted in Stata (version 10, Stata-Corp, TX) and were weighted and adjusted for the complex sample design of the NZHS to make the sample representative of all New Zealand smokers.

**Table 2:** Characteristics of smokers who smoke specified brands ie, who could be classified as "light" or regular smokers for cigarettes or RYO tobacco (n = 1157, with all the results weighted to adjust for the complex sample design and non-response)

**Variable**	**"Lights" smokers (%)**	**Regular smokers (%)**	**Crude odds ratios (*OR*) for being a "lights" smoker (95% *CI*)**
Total (*n *= 1157)	22.9	77.1	-

***Age ***(%)*			
18–24 (*n *= 119)	22.3	77.7	1.00 Referent
25–34 (*n *= 296)	18.8	81.2	0.81 (0.38 – 1.69)
35–44 (*n *= 298)	18.8	81.2	0.81 (0.39 – 1.69)
45–54 (*n *= 240)	27.1	72.9	1.30 (0.62 – 2.72)
55+ (*n *= 204)	30.4	69.6	1.52 (0.74 – 3.11)
***Gender ***(%)*			
Male (*n *= 423)	18.5	81.5	1.00 Referent
Female (*n *= 734)	27.2	72.8	1.64 (1.11 – 2.42)
Ethnicity**			
European (includes other) (*n *= 521)	26.3	73.7	1.00 Referent
Māori (*n *= 532)	15.6	84.4	0.52 (0.35 – 0.77)
Pacific (*n *= 62)	4.1	95.9	0.12 (0.04 – 0.34)
Asian (*n *= 42)	35.1	64.9	1.51 (0.60 – 3.80)
***Deprivation level***(quintiles)*			
1&2 (least deprived) (*n *= 92)	32.1	67.9	1.00 Referent
3&4 (*n *= 172)	23.1	76.9	0.64 (0.31 – 1.32)
5&6 (*n *= 196)	22.3	77.7	0.61 (0.30 – 1.25)
7&8 (*n *= 263)	26.7	73.3	0.77 (0.38 – 1.56)
9&10 (most deprived) (*n *= 434)	16.6	83.4	0.42 (0.21 – 0.84)
***Financial stress & smoking-induced deprivation#***			
*Financial stress*: Unable to pay any important bills on time – "No" (*n *= 1058)	23.7	76.3	1.00 Referent
As above but "Yes" (*n *= 99)	13.3	86.7	0.49 (0.25 – 1.00)
*Smoking-induced deprivation*: Not spending on household essentials – "No" (*n *= 839)	23.1	76.9	1.00 Referent
As above but "Yes" (*n *= 318)	22.6	77.4	0.97 (0.64 – 1.47)

* Based on NZHS data with this collected a few months prior to the ITC Project survey. Deprivation level was based on a New Zealand-specific small area deprivation index, NZDep2006 [30].

** Ethnicity results are based on a prioritised method where all subjects with Māori only or Māori and other ethnic affiliations were classified as Māori; all those with Pacific and other ethnic affiliations were classified as Pacific (unless Māori affiliation was also reported); all those with Asian and other ethnic affiliations were classified as Asian (unless Māori or Pacific affiliation was also reported); and where the European group including those with European ethnicity only and a small number (n = 5) of people with other ethnic affiliations. For more detail, see an online *Methods Report *[30].

# For example "smoking-induced deprivation" was based on the question: "In the last six months, have you spent money on cigarettes that you knew would be better spent on household essentials like food?". For more detail on, see an online *Methods Report *[30].

**Table 3:** Additional smoking behaviour and related beliefs among "lights" and regular cigarette smokers (all the results age-sex adjusted, weighted and adjusted for the complex design)

**Variable**	**"Lights" smokers (column%)** **(n = 247)**	**Regular smokers (column%)** **(n = 910)**	**Adjusted OR for being a "lights" smoker (95% CI)**
***Cigarette consumption***			
Daily smokers	92.2	96.4	1.00 Referent
Weekly/monthly smokers	7.8	3.6	2.31 (1.07 – 4.99)
***Type of tobacco product***			
Factory-made cigarettes only or sometimes smokers	69.8	51.6	1.00 Referent
Only roll-your-own smokers	30.2	48.4	0.49 (0.32 – 0.74)
***Quitting behaviour & intentions***			
Ever tried to quit smoking (no)	42.5	40.5	1.00 Referent
Ever tried to quit smoking (yes)	57.5	59.5	0.92 (0.62 – 1.35)
			
Not planning to quit	22.2	32.2	1.00 Referent
Beyond 6 months	44.2	34.9	2.05 (1.25 – 3.38)
Within next 6 months	24.1	22.9	1.79 (1.02 – 3.16)
Within next month	9.5	10.0	1.45 (0.73 – 2.88)
			
Confident of quitting successfully (% not very sure)	66.6	78.5	1.00 Referent
Confident of quitting successfully (% at least very sure)	33.4	21.5	1.88 (1.24 – 2.84)
***Awareness & beliefs***			
Belief that quitting is not difficult	41.6	30.4	1.00 Referent
Belief that quitting is difficult (at least somewhat)	58.4	69.6	0.58 (0.39 – 0.86)
			
Belief that smoking is no more risky than lots of other things people do (% *not *agreeing)	47.2	44.4	1.00 Referent
As above (but agreeing)	52.8	55.6	0.88 (0.60 – 1.29)
			
Perceived addiction (% not addicted)	12.9	6.5	1.00 Referent
Perceived addiction (% at least somewhat addicted)	87.1	93.5	0.46 (0.23 – 0.92)

**Table 4:** Indices and scales for smoking behaviour and related beliefs of "lights" and regular cigarette smokers

Variable*	**"Lights" smokers** (mean score, 95% CI) (n = 247)	**Regular smokers **(mean score, 95% CI) (n = 910)	**Differences in mean scores** (Test of significance)
Heaviness of smoking index (alternate version)	0.42 (0.06 – 0.79)	1.17 (0.98 – 1.37)	0.75, p < 0.001
Awareness of smoking harm (7-item scale) (α = 0.69)	0.53 (0.46 – 0.60)	0.47 (0.43 – 0.51)	0.06, p = 0.138
Smoking has affected health and quality of life (2-item scale) (α = 0.68)	1.99 (1.84 – 2.14)	2.04 (1.98 – 2.11)	-0.05, p = 0.521
Concern that smoking will lower health and quality of life in the future (2-item scale) (α = 0.78)	2.44 (2.28 – 2.61)	2.43 (2.35 – 2.51)	0.01, p = 0.889
Self-exempting beliefs (3-item scale, high score means stronger such beliefs) (α = 0.60)	2.88 (2.76 – 3.00)	3.02 (2.95 – 3.09)	-0.14, p = 0.043
Intention of quitting (4-point scale, based on questions in Table 3)	1.21 (1.07 – 1.35)	1.11 (1.02 – 1.19)	0.09, p = 0.225
Self-efficacy for quitting (4-point scale)	2.82 (2.57 – 3.06)	2.50 (2.39 – 2.61)	0.32, p = 0.022
Overall attitude to smoking (5-point scale, high score is more positive)	2.40 (2.27 – 2.54)	2.46 (2.39 – 2.53)	-0.06, p = 0.433
Attitude to regulation index (2-item index, high score is favourable toward regulation) (α = 0.51)	3.26 (3.13 – 3.40)	3.58 (3.29 – 3.43)	-0.32, p = 0.221

* For more details of index composition and development, see an online *Methods Report *[30]. Alpha (α) scores are for Cronbach's alpha (see *Methods*).

**Table 5:** Beliefs about "lights" among "lights" and regular cigarette smokers

**Variable**	**"Lights" smokers** **(n = 247)** (column %)	**Regular smokers** **(n = 910)** (column %)	**Adjusted OR# for beliefs by "lights" smokers (95% CI)** (where referent group is those not agreeing)
Agree that "lights" make it easier to quit smoking*	25.4	21.4	1.28 (0.83 – 1.98)
Agree that "lights" are less harmful than regular cigarettes*	41.8	25.6	2.23 (1.48 – 3.36)
Agree that smokers of "lights" take in less tar than smokers of regular cigarettes*	42.8	35.4	1.49 (1.00 – 2.21)
Holding at least one of the above 3 beliefs that "lights" confer health benefits (referent group = those holding none of the above 3 beliefs)	60.4	45.0	2.01 (1.35 – 2.99)
Agree** that "lights" are smoother on the throat and chest than regular cigarettes*	64.8	51.0	1.78 (0.63 – 5.01)
The way a smoker puffs on a cigarette can affect the amount of tar and nicotine a smoker takes in (% true)	58.6	64.5	0.74 (0.51 – 1.09)
The way a smoker holds a cigarette can affect the amount of tar and nicotine a smoker takes in (% true)	24.2	29.7	0.78 (0.52 – 1.17)

* Agree = answered "agree" or "strongly agree" to interview question.

** This question was included only in the first quarter of the Wave 1 survey and subsequently removed to reduce the length of the questionnaire.

# Adjusted by age and sex.

**Table 6:** Logistic regression analyses for associations with reporting being a "lights" smoker*

**Variables**	**Adjusted Odds Ratio (95% CI)**			
	**Model 1** (demo-graphics)	**Model 2** (+ socio-demo-graphics)	**Model 3** (+ smoking behaviour)	**Model 4** (+ smoking beliefs)
***Demographic***				
Age (years)#	1.14 (0.99 – 1.31)	1.13 (0.98 – 1.31)	1.14 (0.98 – 1.32)	1.22 (1.04 – 1.43)
Gender (women vs men)	1.78 (1.20 – 2.65)	1.77 (1.19 – 2.63)	1.57 (1.03 – 2.39)	1.46 (0.96 – 2.24)
Māori vs European	0.53 (0.35 – 0.80)	0.56 (0.37 – 0.84)	0.57 (0.37 – 0.87)	0.55 (0.35 – 0.84)
Pacific vs European	0.14 (0.05 – 0.40)	0.15 (0.05 – 0.44)	0.13 (0.04 – 0.41)	0.11 (0.04 – 0.35)
Asian vs European	2.09 (0.78 – 5.61)	2.15 (0.79 – 5.84)	0.99 (0.42 – 2.35)	0.92 (0.38 – 2.20)
***Socio-demographic***				
Deprivation quintiles (increasing deprivation)	-	0.92 (0.79 – 1.06)	0.94 (0.81 – 1.10)	0.93 (0.79 – 1.09)
***Smoking behaviour***				
RYO smoker (only) vs Others	-	-	0.58 (0.38 – 0.91)	0.57 (0.37 – 0.90)
Heaviness of smoking index (alternate version)** #	-	-	0.86 (0.78 – 0.96)	0.87 (0.78 – 0.96)
***Smoking beliefs***				
Awareness of smoking harm (7-item scale)**	-	-	-	1.82 (1.17 – 2.83)
Holding at least one of the 3 beliefs that "lights" confer health benefits**	-	-	-	1.95 (1.29 – 2.95)

* The aORs in models 2, 3 & 4 are adjusted for the demographic and key socio-demographic variables (ie, deprivation), models 3 & 4 for smoking behaviour variables and model 4 for smoking beliefs. The included variables from the univariate analyses that became not significant in the models (at p < 0.05) were subsequently omitted from the final respective models, except for those considered critical to our conceptual framework (see *Methods*).

** See Tables 3 and 4 for further details on these indices and α scores (for multi-item indices) and Table 5 for the three questions used for the variable in the last row of the above table.

# Continuous variable and for age the actual age (years) was divided by 10 so as to interpret the OR estimates for an increment of 10 years.

## Results

### Sample characteristics

All the results presented below have been weighted to reflect the national population of smokers in New Zealand. This adjusted for the fact that our final sample of interviewed smokers was somewhat dominated by women smokers (61.6%) and older smokers (64.7% of the sample were aged 35 years and over). The most deprived quintile of the total New Zealand population was also over-represented at 36.6% of the sample. The booster sampling used in the NZHS had also resulted in our final sample having disproportionately higher percentages of Māori (44.1%), Pacific (6.5%) and Asian (4.3%) respondents (with the rest being "European/Other" at 45.1%).

In the several months since participating in the NZHS, 12.0% of the total sample had quit smoking, 62.9% were smokers of regular cigarettes (including regular forms of RYO tobacco) and 18.7% were "lights" smokers (Table 1). Out of those who smoked a specific brand, 22.9% were "lights" smokers (Table 2).

### Characteristics of "lights" smokers

**Figure 1:**
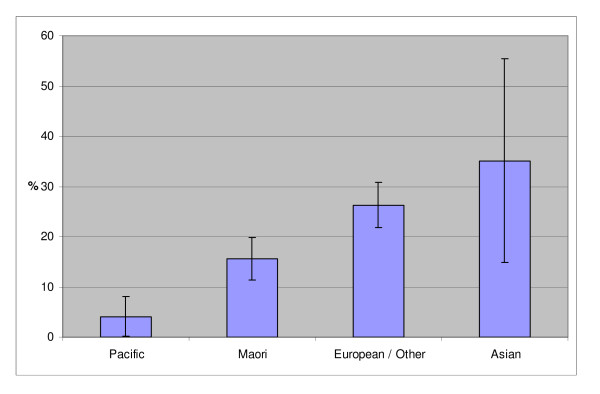
**Proportion of smokers smoking lights by ethnic group**.

For all demographic groups there were more regular than "lights" smokers (Table 2). Compared to smokers of regular cigarettes and tobacco, "lights" smokers were significantly more likely to be older, to be women, and to be of European ethnicity (Table 2). Only 15.6% of Māori, and 4.1% of Pacific peoples were "lights" smokers compared to 26.3% for those of European/Other ethnicity (the 95%CI were very wide for Asians at 14.8% to 55.4%) (Figure 1). Also, "lights" smokers were more likely to reside in a less deprived area (with the proportion of "lights" smokers declining significantly as the level of deprivation increased). The most deprived quintile had only half the proportion smoking "lights" as the least deprived quintile (ie, 16.6% vs 32.1%; odds ratio (OR) = 0.42, 95% CI = 0.21 – 0.84). Smokers who reported evidence of "financial stress" were less likely to smoke "lights" (Table 2).

### Use of, and beliefs about "lights"

"Lights" smokers were more likely to be infrequent smokers (ie, weekly or monthly versus daily) and to have a lower daily consumption of cigarettes (<6 cigarettes per day) (Table 3). The heaviness of smoking index was also under half the value for "lights" smokers compared to regular smokers (Table 4). Similarly, "lights" smokers were only around half as likely to be exclusively RYO smokers (OR = 0.49, 95% CI = 0.32 – 0.74) (Table 3).

"Lights" smokers were more likely to be planning to quit and they were also more confident of being able to quit successfully (Table 3 and the "self-efficacy" scale in Table 4). These beliefs were consistent with their significantly more common perceptions of not being addicted to tobacco and also their more common belief that quitting is not difficult. Yet in terms of actual behaviour, "lights" smokers were not significantly more likely to have made a previous quit attempt.

While "lights" smokers had significantly lower levels of self-exempting beliefs, there were no significant differences between them and regular smokers in terms of "awareness of smoking harm"; the belief that smoking has affected their health and quality of life (or concern for these in the future); overall attitude to smoking; and attitude to regulation to control tobacco smoking (Table 4).

### "Lights" and health

Some of the "lights" smokers had beliefs that "lights" made it easier to quit smoking (25.4%), that "lights" are less harmful (41.8%), and that smokers of "lights" take in less tar (42.8%) (Table 5). Overall most "lights" smokers (60.4%) had at least one of these three beliefs, a proportion significantly higher than for regular smokers at 45.0% (OR = 2.01, 95% CI = 1.35 – 2.99). Belief that "lights" are less harmful was particularly higher amongst "lights" smokers (OR = 2.23; 95% CI = 1.48 – 3.36). Furthermore, more of the "lights" smokers reported that "lights" are smoother on the throat and chest but this difference was not statistically significant.

A majority of both "lights" and regular smokers considered that the way a smoker puffs on a cigarette can affect the amount of tar and nicotine a smoker takes in (58.6% and 64.5% respectively). Only a minority considered that the way a smoker holds a cigarette can affect the amount of tar and nicotine a smoker takes in (24.2% and 29.7% respectively).

### Independent associations between smoker characteristics and smoking "lights"

The probability of being a "lights" smoker independently increased with age and was higher for women (Table 6). Both Māori and Pacific smokers were significantly less likely to smoke "lights" compared to European New Zealanders (ie, for Māori and Pacific respectively in model 1: adjusted OR (aOR) = 0.53, 95% CI = 0.35 – 0.80 and aOR = 0.14, 95% CI = 0.05 – 0.40). In contrast, there was no significant variation by level of deprivation.

These "lights" smokers were also more likely to have lower tobacco consumption (as per the "heaviness of smoking index") and were less likely to be RYO smokers. Having a higher awareness of smoking-related harm was associated with being a "lights" smoker, as was holding at least one of three beliefs that "lights" confer health benefits (aOR = 1.96, 95% CI = 1.29 – 2.96).

## Discussion

### Main findings and interpretation

The key finding from this study is that most smokers of "lights" (60.4%) have at least one of three beliefs in the health benefits of "lights", with this level being statistically significantly higher than for regular smokers (in the multivariate analysis). This indicates that smokers of "lights" appear to be particularly misled by these descriptors, given the lack of scientific evidence for any such health benefits from "lights" (see *Introduction*). Such a finding is compatible with previous work in other countries and for ITC Project countries where this has been studied (see *Introduction*).

Additional findings were supportive of this pattern of beliefs, in that most "lights" smokers (80.0%) reported that "lights" were smoother on the throat and chest (though this was not statistically significantly higher than for regular smokers – Table 5). Most "lights" smokers (58.6%) also reported that how a smoker puffs can affect the intake of tar and nicotine, which is partly true. In contrast, few seemed to understand about vent holes in cigarettes (only 24.2% said that how a cigarette was held could affect tar intake).

While "lights" smokers had significantly lower tobacco consumption and were more "smoking harm" aware, they were still no more likely to be intending to quit or have made a previous quit attempt (ie, the latter two variables were not significant contributors to the models shown in Table 6). These findings are consistent with evidence from elsewhere that smoking "lights" does not facilitate quitting [6,34]. Given that other studies suggest that some smokers believe that smoking "lights" is a step towards quitting [7,11], this appears to be another component of smokers having misperceptions about "lights".

We found no variation in the smoking of "lights" by deprivation (in the multivariate analysis) which is in contrast to the results for higher income being associated with "lights" smoking reported in other English-speaking countries [8]). But our findings of variation in "lights" smoking by ethnicity are novel given that such data have not been reported on in other ITC Project work (eg, in Borland et al [8]) or other previous research. That is we found that both Māori and Pacific smokers were independently *less *likely to smoke lights than European New Zealanders. No reasons for these differences have been suggested in the existing New Zealand literature or our other results from this study, but there may be specific cultural factors that influence brand choice or differing perceptions around value for money. That is although in New Zealand "lights" are priced at the same level as regular cigarettes within each brand grouping, it is possible that some smokers believe they are getting better value for money with regular cigarettes ie, "more taste per dollar spent". Consistent with the idea of cultural and/or price factors was the finding that RYO tobacco smokers were independently *less *likely to smoke "light" forms of loose tobacco. This group may be a particularly price sensitive one and may also have specific attitudes around product naturalness/quality etc [35]. These issues could be further explored in future ITC Project surveys and with qualitative studies.

Other population groups that appear to be the most misled into smoking "lights" according to our results are women and older smokers. Some of these findings are consistent with those found elsewhere (ie, for women [8,11], and older smokers [1,8]). Overall, the use of "lights" in these New Zealand smokers (at 22.9%) was less than that reported elsewhere (eg, a range of 40.1% to 60.4% for four English-speaking countries [8]). This difference may be partly due to the high level of RYO use in New Zealand and the fact that RYO users (according to our data) are less likely to smoke RYO brands that are branded as "light".

### Limitations of this study

As for all such studies a limitation is reliance on smokers' self-report and recall. Possibly in a future NZHS the actual cigarette packets used by the smoker could be observed (or even photographed) by the interviewer. Furthermore, some people did not report a routine brand (Table 1) and it is possible that there is a group of smokers who profess a regular brand but who actually brand switch quite frequently. Indeed, 14% of the sample reporting a regular brand actually said that they smoke both factory-made cigarettes and RYO tobacco regularly. Anecdotal reports to members of our research team from New Zealand shop keepers also suggest that since the launch of the pictorial health warnings in New Zealand in 2008, some smokers even switch brands in the shop (ie, to avoid those packets that have new graphic health warnings or particular warnings they dislike most).

We also suspect that New Zealand smokers might display some social desirability bias in their responses to surveys (eg, possibly being more likely to report having made past and recent quit attempts etc). This is because smoking is becoming increasingly denormalised, as shown by reductions in socially-cued smoking with the recent expansion of smokefree environment laws [36]. But despite these issues, we consider that this study provides reasonably reliable evidence for the use and beliefs around "lights" and variation in use by ethnicity, deprivation and other smoker characteristics.

### Policy implications

An international response to smokers being misinformed about the health "benefits" of "lights" has already started with the requirements of the Framework Convention on Tobacco Control (FCTC) (see *Introduction*). However, just banning light and mild descriptors may not be enough, given the evidence from the European Union ban in the UK setting [13]. This is because such beliefs may be deeply held and are reinforced by "the use of reassuring terms, images and colouring in product marketing" [13].

A recent review has indicated that a more comprehensive solution is to require plain packaging [37]. These authors stated that: "Requiring plain packaging is consistent with the intention to ban all tobacco promotions. There is no impediment in the FCTC to interpreting tobacco advertising and promotion to include tobacco packs". Plain packaging has been defined as requiring a brand name "in a standardized font, size, colour, and location" with no brand logos, colours and corporate symbols [38]. Except for the name, tobacco packs would have a uniform colour, shape, size and texture. Future revisions to FCTC provisions could therefore more strongly indicate the desirability of plain packaging.

It has also been suggested that to counter the decades of misleading marketing about low tar and filtered cigarettes, tobacco product manufacturers should be required to support a remedial public education campaign to inform consumers about important facts, including the presence and function of filter vents, the use of additives and other technologies used in fabricating "low-tar" cigarettes, the contaminants that may be present in the combustible column and on the filter tip, the constituents of smoke that may be hazardous, and the addictiveness of nicotine [10]. To ensure that such campaigns are effective, the campaign content would need to be tightly controlled by government health and social marketing agencies (while still potentially being fully funded by the tobacco product manufacturers).

Our results, that most "lights" smokers reported that "lights" are smoother on the throat and chest, has been described by others [8]. It has been argued that the experience of smokers that "lights" seem less harsh when smoked may confirm their belief that these cigarettes are less harmful, and their belief that they are taking appropriate steps to protect their health [1]. This suggests that as well as the steps detailed above, health regulators could act to ensure that all cigarettes are designed so that none convey the impression of the smoke being less harsh than others. This could involve bans on filter vents [13] and possibly banning various additives (eg, menthol). Of course, another alternative is to move towards banning tobacco products entirely (as articulated by some Māori politicians in New Zealand [39]) and to replace them with a range of pharmaceutical-grade nicotine products.

## Conclusion

In this national sample most "lights" smokers were found to have one or more misperceptions about the product they use, and were no more likely to intend to quit or to have made a quit attempt. This finding is consistent with the international literature. In response to such misperceptions, governments could act further to eliminate all misleading tobacco marketing. Ideally, they could not only adopt FCTC requirements, but go further by requiring plain packaging for all tobacco products.

## Competing interests

The authors declare that they have no competing interests.

## Authors' contributions

NW, GT and RE established the NZ-arm of this study and contributed to design changes (relative to the 4-country ITC Project). JP and MD participated in survey management and with data quality issues. DW undertook all of the statistical analyses but with input from NW and RE. NW and JP did most of the work on drafting the manuscript but all authors contributed to the manuscript and approved the final manuscript.

## Pre-publication history

The pre-publication history for this paper can be accessed here:


